# A 3-gene signature comprising CDH4, STAT4 and EBV-encoded LMP1 for early diagnosis and predicting disease progression of nasopharyngeal carcinoma

**DOI:** 10.1007/s12672-023-00735-x

**Published:** 2023-07-01

**Authors:** Shu-Chen Liu, Chun-I Wang, Tzu-Tung Liu, Ngan-Ming Tsang, Yun-Hua Sui, Jyh-Lyh Juang

**Affiliations:** 1 Department of Biomedical Sciences and Engineering, National Central University, 300, Zhongda Rd., Jhongli Dist., Taoyuan City, 320317 Taiwan; 2 Department of Biochemistry, School of Medicine, China Medical University, Taichung, Taiwan; 3 Department of Radiation Oncology, China Medical University Hsinchu Hospital, Zhubei City, Hsinchu County Taiwan; 4 Institute of Molecular and Genomic Medicine, National Health Research Institutes, Zhunan, 35053 Miaoli County Taiwan

**Keywords:** Nasopharyngeal carcinoma, CDH4, STAT4, CYLD, EBV-encoded LMP1

## Abstract

**Purpose:**

Nasopharyngeal carcinoma is highly metastatic but difficult to detect in its early stages. It is critical to develop a simple and highly efficient molecular diagnostic method for early detection of NPC in clinical biopsies.

**Methods:**

The transcriptomic data of primary NPC cell strains were used as a discovery tool. Linear regression approach was used to define signatures distinctive between early and late stage of NPC. Expressions of candidates were validated with an independent set of biopsies (n = 39). Leave-one-out cross-validation technique was employed to estimate the prediction accuracy on stage classification. The clinical relevance of marker genes was verified using NPC bulk RNA sequencing data and IHC analysis.

**Results:**

Three genes comprising CDH4, STAT4, and CYLD were found to have a significant differentiating power to separate NPC from normal nasopharyngeal samples and predicting disease malignancy. IHC analyses showed stronger CDH4, STAT4, and CYLD immunoreactivity in adjacent basal epithelium compared with that in tumor cells (*p* < 0.001). EBV-encoded LMP1 was exclusively expressed in NPC tumors. Using an independent set of biopsies, we showed that a model combining CDH4, STAT4, and LMP1 had a 92.86% of diagnostic accuracy, whereas a combination of STAT4 and LMP1 had a 70.59% accuracy for predicting advanced disease. Mechanistic studies suggested that promoter methylation, loss of DNA allele, and LMP1 contributed to the suppressive expression of CDH4, CYLD, and STAT4, respectively.

**Conclusion:**

A model combining CDH4 and STAT4 and LMP1 was proposed to be a feasible model for diagnosing NPC and predicting late stage of NPC.

**Supplementary Information:**

The online version contains supplementary material available at 10.1007/s12672-023-00735-x.

## Introduction

Nasopharyngeal carcinoma (NPC) is a highly metastatic malignancy. Despite recent advances in treatment approaches, the prognosis remains poor in advanced NPC, which may be caused by delayed diagnosis. Since the WHO type II and III NPC tumors harbor clonal Epstein-Barr virus (EBV) genomes, EBV nucleic acids are considered as a potential prognosis marker for NPC. For example, the plasma EBV-DNA levels from advanced-stage NPC patients were found significantly higher than that from early-stage patients [1–3]. However, EBV itself does not become a tumor-specific biomarker for the diagnosis of NPC. This viral infection is also associated with other types of malignancies, including Hodgkin’s lymphoma, post-transplant lymphoma, hairy leukoplakia, Burkitt’s lymphoma, and others [4]. Thus, it is critical to develop a simple and efficient auxiliary diagnosis method for early detection of NPC in clinical biopsies.

Several EBV latent infection proteins, such as latent membrane proteins (LMP1, LMP2A and LMP2B) and EBNA1 have been demonstrated to associate with the pathogenesis of undifferentiated NPC [4]. The EBV-encoded LMP1 is a key viral oncoprotein in the pathogenesis of undifferentiated NPC and was frequently detected in clinical NPC biopsies [5]. Interestingly, for the EBV-driven tumorigenesis, the C-terminal activation regions (CTARs) of LMP1 can activate selective STAT family proteins, such as STAT3, STAT5, and STAT1 [6, 7], but not STAT4. Chen et al. have demonstrated that STAT4 is capable of binding to LMP1 promoter, however, unlike the oncogenic role of activated (nuclear) STAT3 in EBV-driven tumorigenesis, STAT4 expression is relatively low and restricted in the cytoplasm of NPC tumors [8]. The Cylindromatosis (CYLD) gene is located on 16q12-13 and loss of CYLD alleles has been reported to be one of the causes of cylindroma [9]. CYLD alterations have been detected in several types of human cancers, including colon cancer, hepatocellular carcinomas [10], melanoma [11], and head and neck cancers [12, 13]. It functions as a deubiquitinating enzyme, which negatively regulates TRAF2 and NF-kB signaling pathway [14, 15]. The NF-kB pathway is well known for its involvement in cell survival and oncogenic transformation as well as immune responses. Cadherin 4 (CDH4) is a member of the cadherin family encoding for the retinal cadherin (R-cadherin). CDH4 deficiency has been reported in several types of cancer, such as gastrointestinal tumors [16] and lung cancers [17]. Miotto et al. have showed that CDH4 genomic sequence contains CpG-dense islands in the promoter region, in which hypermethylation is frequently observed in human colorectal and gastric carcinomas [16]. CDH4 is therefore considered as a tumor suppressor gene.

One of the major underlying causes of treatment failure in EBV-associated NPC is the high incidence of local recurrence and metastasis (~ 20%), and the progression of NPC is profoundly affected by EBV infection. However, it is difficult to detect NPC in its early stages. Therefore, the development of a simple and highly efficient molecular diagnostic method can serve as a parallel approach to histology for NPC early diagnosis and predicting disease malignancy with biopsy samples. This study provides strong evidence that a 3-gene signature model, combining CDH4 and STAT4 and EBV-encoded LMP1, has a significant differentiating power to separate NPC from normal nasopharyngeal samples and predicting disease malignancy.

## Materials and methods

### Clinical samples

A total of sixty-six pretreatment NPC biopsy samples and fifty-three nasopharyngeal biopsy samples from normal donors were collected from Tissue bank, Chang Gung Memorial Hospital, Taiwan. All NPC tumors were histologically confirmed by pathologists. Thirty-nine NPC biopsies fixed in formaldehyde were used for immunohistochemical verification of candidate gene expressions. Eighteen NPC biopsies and twenty-one normal nasopharyngeal biopsies were used as an independent validation set to test the predictive accuracy for NPC diagnosis by QRT-PCR assay. Prior informed consent was obtained from all participants for the use of these materials for research study.

### Cell culture

Primary nasopharyngeal carcinoma cells and primary normal nasopharyngeal epithelial (NPE) cells were isolated from fresh human biopsies. Briefly, nasopharyngeal biopsies were trimmed into 1–2 mm explants and distributed on the top of mitomycin C-treated NIH/3T3 feeder layers in DMEM medium supplemented with 10% FBS (Gibco), 10 µg/mL gentamicin (Invitrogen) and 2 µg/mL amphotericin B (Sigma-Aldrich). As epithelial outgrowths from the explants were visualized, cells were fed with defined keratinocyte serum-free medium (Gibco) to stimulate proliferation of epithelial cells. Five NPC-derived cell lines: CNE1, CNE2, NPC-TW01, NPC-TW06 and HONE cells [18–21] were cultured in DMEM medium supplemented with 10% FBS. HK1 NPC cells were grown in PRMI-1640 medium supplemented with 10% FBS. NPC-derived cell lines were authenticated via 16 core short tandem repeat (STR) locus profiling (analyzed by Bioresource Collection and Research Center, Taiwan).

### Quantitative real-time reverse transcription-polymerase chain reaction (QRT-PCR)

Total RNA was prepared with RNeasy mini kit (QIAGEN). One microgram total RNA was used to synthesize cDNA using the First Strand cDNA Synthesis Kit (Roche). Diluted cDNA was used for QRT-PCR with FastStart DNA Master SYBR Green I kit (Roche) and LightCycler 96 system (Roche) following the manufacturer’s protocol. Primers (Supplementary Table S1) used in this study were designed using LightCycler probe design software. Expression was expressed relative to that of GAPDH. All assays were conducted in triplicate.

### Immunofluorescence

Cells were grown on sterile glass coverslips and fixed with 4% paraformaldehyde in PBS, followed by permeabilization with 0.3% Triton X-100 and blocking with 8% normal goat serum. Cells were subjected to immunostaining using primary antibodies against human CDH4 (sc-398306, Santa Cruz Biotechnology), CYLD (PA5-34630, Invitrogen), and STAT4 (ab68156, Abcam), and visualized with Alexa Fluor-conjugated secondary antibodies (Invitrogen). Nuclei were stained with Hoechst 33342 (Invitrogen).

### Gene discovery for NPC specific signatures and stage classification

To identify NPC-specific markers and genes that can best discriminate between the early- and late- stage patients, we used linear regression approaches to select markers from transcriptomic data comprising 9 primary NPC cell strains versus a pool of 32 normal nasopharyngeal epithelial cell strains, which served as a universal reference (GSE14262) [22]. We used Mpg to denote the normalized log_2_ intensity ratio for gene g of patient p, whereas g = 1,…,35,185, and p = 1,…,9. Let Xp denote the TNM stage for patient p with Xp = 1 indicating early stage and Xp = 0 late stage. For each gene g, we fit a simple linear regression of the normalized log intensity ratio on TNM stage, and denoted ag the estimated intercept and bg the estimated slope, respectively. Let S1, S2, and S3 be the standard deviation of the set {ag}, {bg} and {ag + bg} respectively, and let Q1(q) and Q2(q) be the 100q% quantile of the set {ag} and {bg}, respectively. We selected genes belonging to the following 4 groups:

C_I _= {g: ag > 2S1, bg < Q2(0.2), ag + bg<−2S3},

C_II _= {g: ag<−2S1, bg > Q2(0.8), ag + bg > 2S3},

C_III _= {g: ag < Q1(0.1), |bg|<0.1S2},

C_IV _= {g: ag > Q1(0.9), |bg|<0.1S2},

Genes in group I and II are likely to exhibit a reverse expression pattern able to discriminate early and late-stage NPC samples, whereas genes in group III and group IV are likely to be consistently down- or up-regulated across all NPC patients compared with normal donors. There are 17 genes in C_I_, 82 genes in C_II_, 187 genes in C_III_, and 384 genes in C_IV_ (Supplementary Table 2).

### Leave-one-out cross validation for predicting early or late stage of NPC

To evaluate genes in C_I_ and C_II_ were representative in predicting early or late stage of NPC, we employed leave-one-out cross-validation analysis to estimate the prediction accuracy on stage classification. For each gene g, we calculated the difference in averaged log_2_ intensity ratio Dg between early and late stage; i.e. Dg = Mg-M′g where Mg and M′g is the sample mean of the set {Mpg: Xp = 0} and {Mpg: Xp = 1}, respectively. Genes with high D values indicate high discriminating power between early and late stage. We selected genes with top 5% D value as potential candidates able to discriminate early and late stage for diagnostic purpose. For a new patient with log_2_ intensity ratios {Ng}, we calculated d1 the Euclidean distance between {Ng} and {Mg} for selected candidate genes and d2 Euclidean distance between {Ng} and {M′g} for selected candidate genes. If d1 > d2, then we classified the new patient’s TNM stage as early stage, otherwise, the late stage. Overall, a 100% prediction accuracy was obtained.

### Establishment of prediction models

We built models based on expressions of three cellular genes, including CDH4, STAT4 and CYLD, as well as EBV-encoded LMP1 in NPC biopsy samples using QRT-PCR assays. The cellular genes were applied in the discriminant analysis to screen out potential NPCs. The expression of LMP1 was used as a marker for the assignment of NPC. Because of the difficulty of obtaining fresh NPC biopsies to increase sample size, four-fold cross-validation was used in this study. The nasopharyngeal biopsies were randomly separated into two subsets, the initial subset with 21 samples served as a training dataset; the other subset was the testing dataset. This procedure was repeated for a thousand times in order to assess performance of the models.

### Immunohistochemistry

Pretreatment formalin-fixed paraffin-embedded (FFPE) NPC tumor tissues were collected to examine protein expression (approved by the Institutional Review Board of Chang Gung Memorial Hospital, Taiwan). Immunohistochemistry was performed using Leica BOND-MAX system and the Bond Polymer Refine Detection Kit (Leica Microsystems, DS9800) as described previously [23]. The primary antibodies used were as follows: mouse anti-human CDH4 (1: 50; sc-398306, Santa Cruz Biotechnology, Inc.), rabbit anti-human CYLD (1:100; PA5-34630, Invitrogen), and rabbit anti-human STAT4 (1:100; ab68156, Abcam). For the negative control, the primary antibody was omitted and replaced with blocking buffer containing the same amount of IgG from non-immune rabbit or mouse serum. The staining score was defined on a 0 to 3 scale according to staining intensity and extent (score = 0: negative or weak staining in 15% of epithelium/tumor cells; score = 1, weak staining in 15–50% of epithelium/tumor cells; score = 2, weak staining in more than 50% of cells or moderate staining in less than 50% of cells; 3, moderate staining in 50–70% of cells or strong staining in less than 70% of cells). The IHC results were reviewed by pathologists.

### 5-Aza-2′-dC treatment

To investigate whether the reduced expression of candidate genes can be reversed by demethylating agents. NPC-derived cell lines: CNE1, CNE2, NPC-TW06, and HONE1 were tested for this purpose. HeLa cells was used as a positive control [16] and primary human fibroblasts (NPF cells) were served as a normal cell control. Cells were seeded at a density of 1 × 10^5^/mL and cultured for 24 h, then treated with 1 µM of 5-Aza-2′-dC (Sigma Aldrich) for four days. Medium and the drug were replaced every 24 h. Total RNAs were extracted after treatment and the expression was analyzed by gel-based RT-PCR assay. Uncut blots were summarized in Supplementary Figure S1.

### Measurement of DNA copy number change

Genomic DNA was extracted using the DNA Extraction kit (QIAGEN) according to the instructions of the manufacturer. DNA copy number changes were measured by quantitative real-time PCR assays. The genomic sequence of CYLD gene was retrieved from Ensembl database (ENSG00000083799). Two pairs of PCR primers were designed to amplify genomic DNA regions from 49343023 to 49343257 and 49375578 to 49375771, respectively. Each amplicon contains region across an exon-intron boundary. Quantitative PCR results were normalized to Sialyltransferase 4 A (SIAT4A) gene, which was identified to be presented in equal DNA copy number for all samples investigated in this study. All values measured were compared to normal nasopharyngeal epithelial strains. DNA copy number loss was defined as normalized ratio < 0.8 and DNA amplification as > 1.2.

### Cell transfection

NPC-TW01cells and NPC-TW06 cells were seeded at a density of 1 × 10^5^ cells/well of a 6-well plate 24 h prior to transfection with either 0.5 µg of empty pSG5 vector or equal amount of LMP1 expression plasmids (pSG5_LMP1) [24] using Fugene HD transfection reagents (Promega). Total RNA and protein lysates were harvested 36 h post-transfection.

### Statistical methods

Statistical analyses were performed using GraphPad Prism 5 (GraphPad Software). IHC analyses of immunoreactivity in human NPC biopsies were evaluated using the chi-square test. All statistical tests were two-sided and *p* values < 0.05 were considered statistically significant.

## Results

### Transcriptomic analysis defines expression signatures distinctive between early and late stage of NPC

**Fig. 1:**
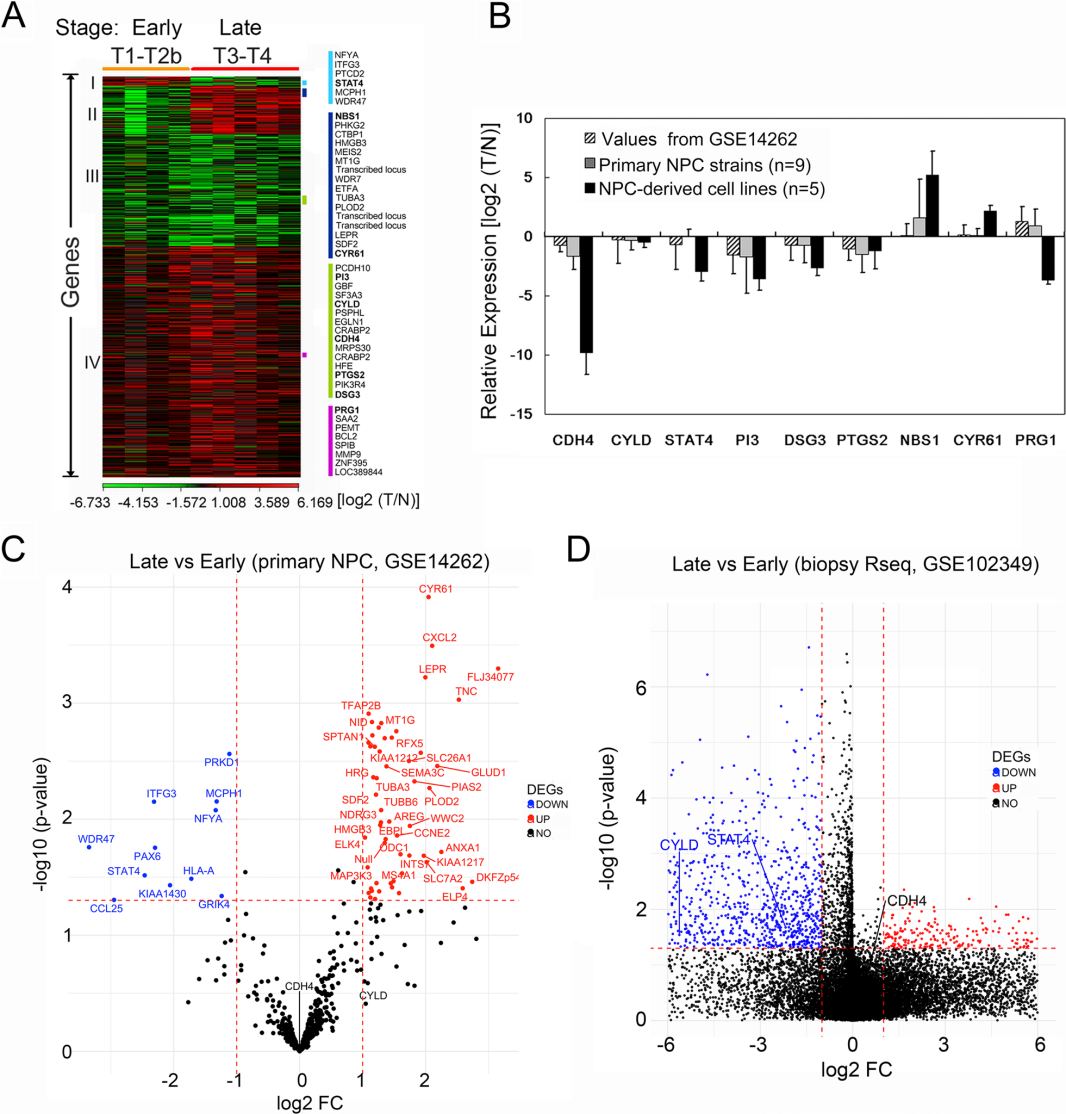
Target genes discovery and validation. **A** heatmap showed four clusters analyzed by linear regression approach. Transcriptomic data were extracted from Gene Expression Omnibus (GSE14262). Rows represent individual genes and columns represent individual NPC samples arrayed according to stage. A positive fold change (red) indicates increased expression and a negative fold change (green) indicates decreased expression in NPC samples compared to the normal controls. **B** validation of candidate genes by QRT-PCR in nine primary cultured NPC samples that had served for the transcriptomic analysis and in five NPC-derived cell lines. mRNA expression was normalized to that of GAPDH. Ratio of expression was log_2_ transformed for QRT-PCR and transcriptome data. Values are presented as mean and SD of triplicate. T, tumor; N, a mixture of 32 primary normal nasopharyngeal epithelial strains. **C**, **D** Volcano plots revealed differentially expressed genes (DEGs). Cutoff values were defined as |log2(FC)| > 1 and a *p* value < 0.05. Transcriptomic data were extracted from GEO database

To identify NPC-specific molecular signatures and genes that can best discriminate between the early- and late- stage patients, we used linear regression approach to select classifier genes from ~ 35,000 informative genes (GSE14262) [22]. A total of 670 genes were extracted and grouped into four clusters (Fig. 1A and Supplementary Table 2). The expression variation of genes in Cluster I and II revealed a distinct pattern between early and late stage of NPC. Genes in Cluster I (17 genes) were uniquely downregulated in late stage of NPC, whereas genes in Cluster II (82 genes) were overexpressed in advanced NPC. In addition, among the 670 genes, 187 were consistently downregulated in NPC samples (Cluster III). On contrary, 384 genes involved in Cluster IV were overexpressed in virtually all NPC samples investigated in this study. To look for clinically useful NPC markers based on QRT-PCR assay, which is a relatively simple approach for diagnostic purpose, we selected nine genes from Cluster I to IV. The selected genes in Cluster I (STAT4) and Cluster II (NBS1 and CYR61) were for distinguishing advanced NPC from early stage of NPC, whereas genes chosen from Cluster III (CDH4, CYLD, PI3, DSG3, and PTGS2) and Cluster IV (PRG1) were consistently dysregulated across all NPC samples investigated. Among these candidates, CDH4, CYLD, STAT4 were markedly downregulated in primary NPC cell strain as well as in five NPC-derived cell lines compared with a pool of 32 normal nasopharyngeal epithelial cell strains (Fig. 1B). Volcano plots of NPC transcriptomes derived from GEO database further revealed marked downregulation of STAT4 in the late stage of NPC compared to the early- stage tumors (Fig. 1C, D).

### Expression of CDH4, STAT4, and CYLD in primary NPC cells

**Fig. 2:**
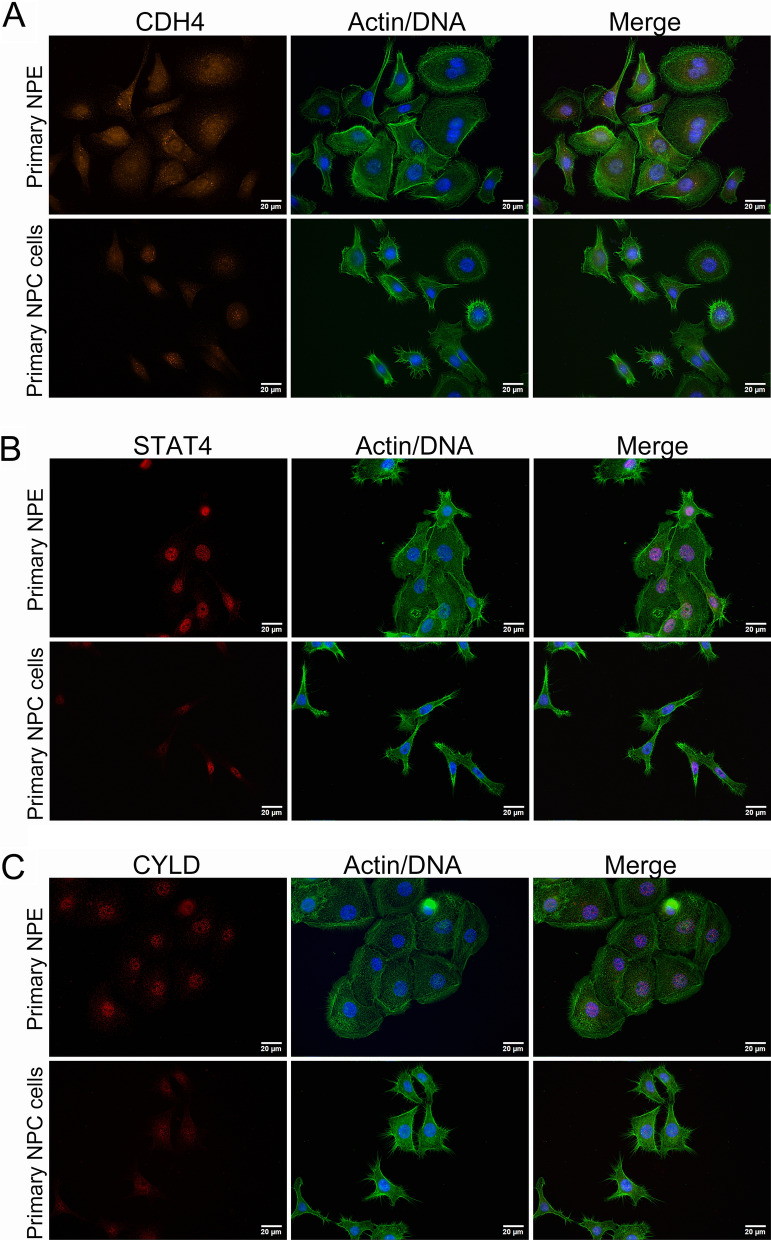
Immunostaining of CDH4, STAT4, and CYLD in primary nasopharyngeal cells and normal nasopharyngeal epithelial cells. **A** CDH4 expression. **B** STAT4 expression. **C** CYLD expression. NPE: primary nasopharyngeal epithelium isolated from normal nasopharyngeal biopsies. Alexa Fluor 488 phalloidin (green) was used to stain F-actin. Blue, nuclear staining. Scale bar, 20 μm

With an attempt to develop a simple quantitative PCR-based assay for clinical application, we examined whether the mRNA expressions of CDH4, STAT4, and CYLD were consistent with the protein levels expressed in primary NPC cells and normal NPE cells isolated from biopsies of NPC patients and patients diagnosed with sinusitis, respectively. Results of immunocytochemical analysis confirmed that the protein expressions of CDH4 (Fig. 2A), STAT4 (Fig. 2B), and CYLD (Fig. 2C) were markedly downregulated in primary NPC cells compared with NPE cells. These candidates were then tested to evaluate the potential for molecular diagnosis of NPC.

### The applicability of selected molecules in clinical biopsy samples

**Fig. 3:**
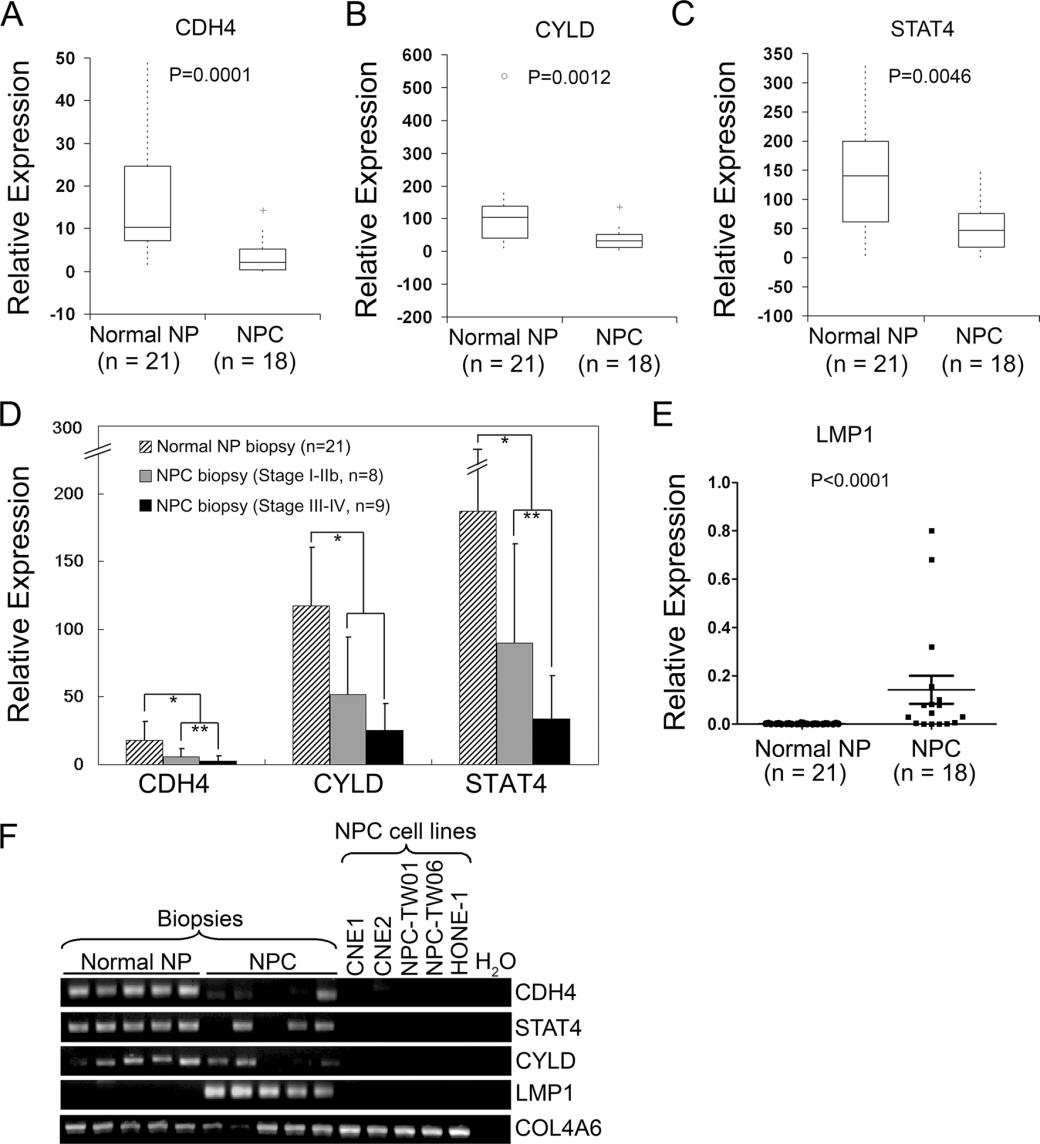
Expression analysis of CDH4, CYLD, and STAT4 in human NPC biopsy samples. **A** CDH4 expression. **B** CYLD expression. **C** STAT4 expression. mRNA expressions were measured by QRT-PCR assays. Values of box plots are presented as mean and SEMs. **D** Comparison of marker gene expression among samples diagnosed with either early (stage I-IIb, n = 8) or late stage (stage III-IV, n = 9) of NPC. *p < 0.05, **p < 0.01, ***p < 0.001 (Mann-Whitney test). **E** Expression of EBV-encoded LMP1 in biopsy samples. All expression levels were normalized to that of GAPDH. **F** Agarose gel electrophoresis of the RT-PCR products. Three groups of samples were examined: normal nasopharyngeal biopsies, NPC biopsies, and five NPC-derived cell lines. COL4A6 was amplified in the same PCR sample as a control gene. Uncut blots for the gel-based PCR assay were summarized in Supplementary Figure S1

To determine whether these candidate markers identified from primary cell strains could be applicable to the biopsy samples, which contain heterogeneous populations. The expression of CDH4, STAT4, and CYLD were investigated in an independent biopsy sample set, including 18 NPC biopsies and 21 normal nasopharyngeal biopsies using QRT-PCR analysis. Results showed a significantly differential expression of CDH4 (Fig. 3A), CYLD (Fig. 3B) and STAT4 (Fig. 3C) between NPC biopsies and normal nasopharyngeal biopsies. In addition, the reduced expression of CDH4 and STAT4 also appeared to correlate with late stage of NPC (*p* < 0.01) (Fig. 3D). The EBV-encoded LMP1 was also used to confirm the collected NPC biopsies are tumor origin. LMP1 expression was uniquely detected in tumor biopsies analyzed either by QRT-PCR assay (Fig. 3E) or gel electrophoresis of the cDNA PCR products (Fig. 3F). These results suggest that CDH4, STAT4, and CYLD were potential cellular markers for RT-PCR-based analysis of NPC biopsy samples, and LMP1 might act as a supporting marker for the diagnosis.

### Predictive accuracy of a three-gene model for disease diagnosis and tumor malignancy

To further evaluate the prediction power of candidate biomarkers that have greater likelihoods of becoming clinically useful markers for NPC diagnosis and for monitoring tumor progression, we built multiple logistic regression models considering the expression of CDH4, STAT4, CYLD and LMP1. We calculated the predictive accuracy rate using leave-one-out cross validation method based on mRNA expression detected in biopsy samples. For disease diagnosis, the composition of (CDH4 + LMP1) or (CDH4 + STAT4 + LMP1) resulted in a best predictive accuracy rate (92.86%) (Table 1). A minimum of 71.43% accuracy rate was obtained for any composite model in terms of NPC diagnosis. For monitoring tumor malignancy, STAT4 alone or (STAT4 + LMP1) could acquire an equally higher accuracy rate (70.59%) (Table 1). Collectively, the composition of CDH4, STAT4, and LMP1 constitutes an ideal model for disease diagnosis as well as for monitoring tumor malignancy.

**Table 1:** Predictive accuracy of logistic regression models for NPC diagnosis and predicting tumor malignancy

Model	Prediction accuracy (%)^a^	
	NPC diagnosis^b^	Tumor malignancy^c^
CDH4	71.43	58.82
CYLD	82.14	58.82
STAT4	75.00	70.59
LMP1	75.00	58.82
CDH4 + CYLD	75.00	52.94
CDH4 + STAT4	75.00	58.82
CDH4 + LMP1	92.86	52.94
CYLD + STAT4	78.57	64.71
CYLD + LMP1	85.71	52.94
STAT4 + LMP1	85.71	70.59
CDH4 + CYLD + STAT4	71.43	58.82
CDH4 + CYLD + LMP1	85.71	52.94
CDH4 + STAT4 + LMP1	92.86	58.82
CYLD + STAT4 + LMP1	85.71	58.82
CDH4 + CYLD + STAT4 + LMP1	82.14	47.06

^a^Accuracy rate was measured by leave-one-out cross validation based on quantitative real-time PCR values of candidate genes

^b^A total of 39 nasopharyngeal biopsy samples were used, including 18 NPC biopsies and 21 normal nasopharyngeal biopsies

^c^NPC biopsies were divided into two groups according to TNM stage, stage I–II_b_ were defined as early stage (n = 8), whereas stage III–IVa were classified as late stage (n = 9)

### Clinical relevance of marker gene expression in NPC biopsies

**Fig. 4:**
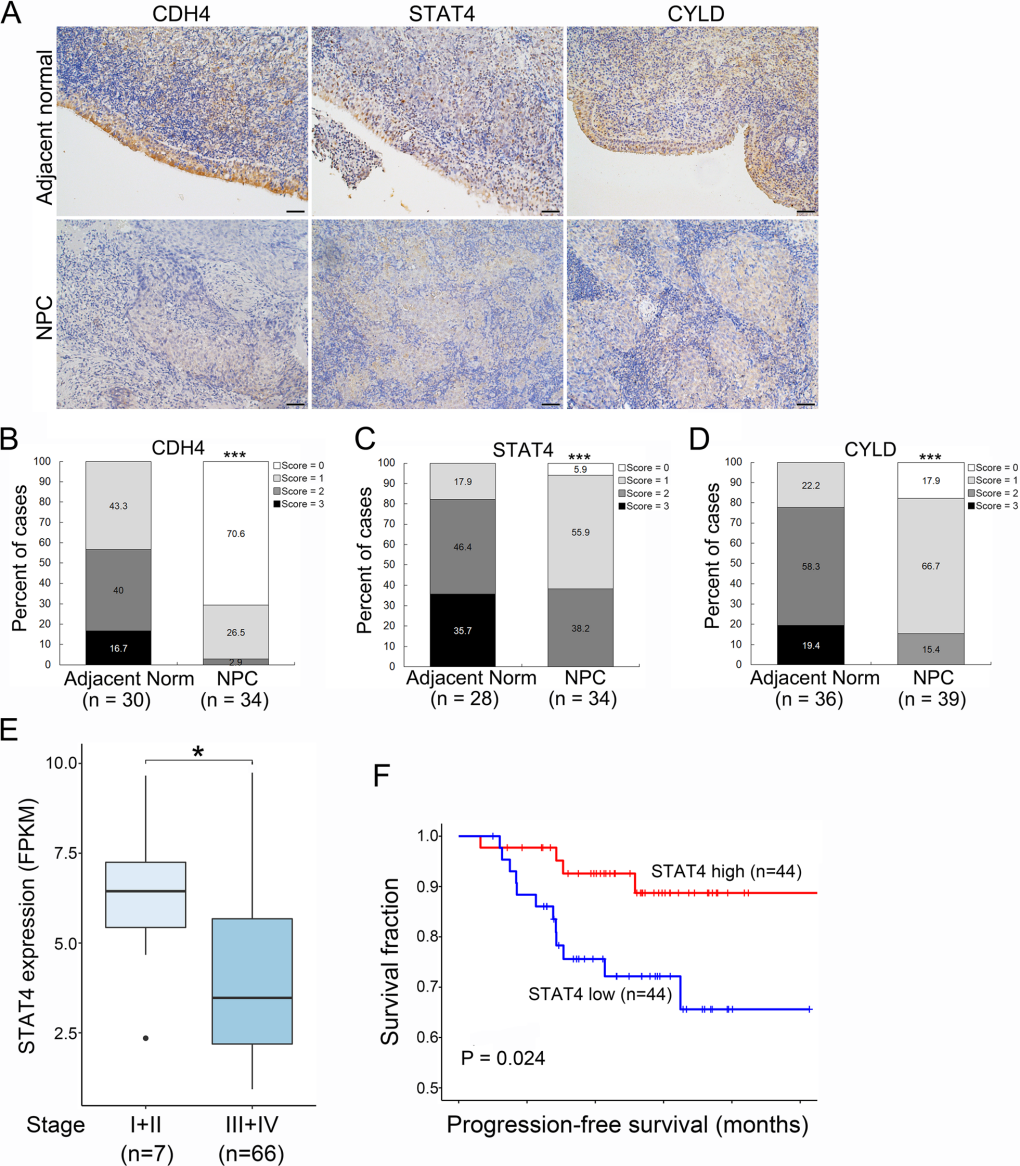
Clinical relevance of CDH4, STAT4, and CYLD in NPC. **A** Representative images of CDH4, STAT4, and CYLD immunoreactivities in adjacent normal epithelium and NPC tumor tissues. Scale bar, 50 μm. **B**–**D** statistical analysis of CDH4 (**B**), STAT4 (**C**), and CYLD (**D**) in NPC tumor tissues. ***p < 0.001 (chi-square test). **E** Reduced expression of STAT4 in late stage of NPC. Data were extracted from GEO database (GSE102349). *p < 0.05 (Mann–Whitney U test). **F** Kaplan–Meier progression-free survival curves of NPC patients based on STAT4 levels. Data were extracted from GEO database (GSE102349).

To evaluate the protein levels of CDH4, CYLD, and STAT4, we conducted immunohistochemical analysis on paraffin-embedded NPC tissue sections. The staining intensity of adjacent basal epithelial cells within the tumors was served as a control. IHC results demonstrated that the expression levels of CDH4, CYLD, and STAT4 were stronger in adjacent basal epithelium compared to that in tumor cells (*p* < 0.001) (Fig. 4A–D). Of the thirty-four NPC evaluated, CDH4 tumor expression were negative in twenty-four (70.6%) cases, nine (26.5%) were score = 1, and one (2.9%) was score = 2, whereas thirteen (43.3%) of the cases were score = 1, twelve (40.0%) were score = 2, and five (16.7%) were score = 3 in adjacent nasopharyngeal epithelium (Fig. 4B). STAT4 levels were two (5.9%) negative expression, nineteen (55.9%) were score = 1, and thirteen (38.2%) were score = 2 in NPC tumors, whereas five (17.9%) were score = 1, thirteen (46.4%) were score = 2, and ten (35.7%) were score = 3 in adjacent nasopharyngeal epithelium (Fig. 4C). The immunoreactivities of CYLD were seven (17.9%) negative, twenty-six cases (66.7%) were score = 1, and six (15.4%) were score = 2 in NPC tumors, whereas eight (22.2%) cases were score = 1, twenty-one (58.3%) were score = 2, and seven (19.4%) were score = 3 in adjacent nasopharyngeal epithelium (Fig. 4D). Collectively, the immunoreactivities of CDH4, STAT4, and CYLD were markedly decreased in tumors compared to adjacent basal epithelium (*p* < 0.001, chi-square test). The IHC data demonstrated that mRNA transcripts of CDH4, STAT4, and CYLD corresponded well with their protein expressions in biopsy samples. Analytical results on a large-scale NPC RNA sequencing dataset (GSE102349) showed that suppressed STAT4 expression was not only evident in late stage of NPC (Fig. 4E), also correlated with poorer progression-free survival of NPC patients (Fig. 4F).

### Promoter hypermethylation, DNA copy number change, and EBV-LMP1 contribute to the reduced expression of CDH4, CYLD, and STAT4, respectively

**Fig. 5:**
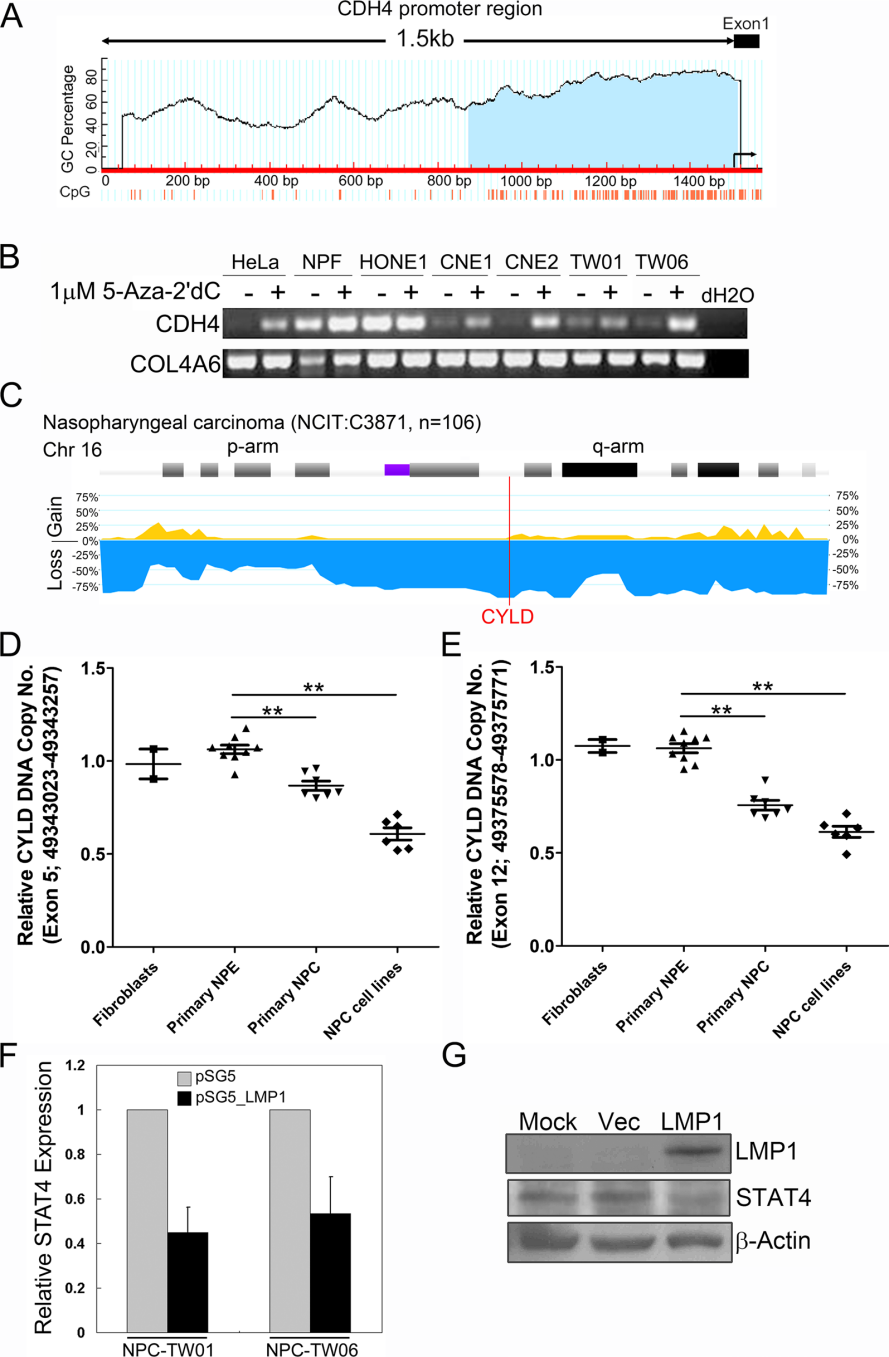
Potential mechanisms contribute to the suppressive expression of CDH4, CYLD, and STAT4 in NPC. **A** Dense CpG islands present at the CDH4 promoter region. The vertical bars indicate the CpG dinucleotides present in the region. **B** Demethylation treatment restored the expression of CDH4 in NPC cell lines. Expression was measured by gel-based reverse transcription-PCR assay. **C** Analysis of CYLD DNA copy number changes in NPC biopsies. Data were extracted from the progenetix cancer genome database (NCIT: C3871). **D**, **E** CYLD DNA copy number analysis. Quantitative real-time PCR assays were conducted to detect copy number changes in primary NPC cell strains, primary normal nasopharyngeal epithelial strains, NPC-derived cell lines, and fibroblasts isolated from NPC biopsy. Two pairs of primer were designed to amplify the CYLD genomic DNA regions: 49343023 to 49343257 (**D**) and 49375578 to 49375771 (**E**). Data are presented as means and SEMs. **F** Effect of EBV-encoded LMP1 on STAT4 expression in nasopharyngeal cells. NPC cells were transiently transfected with a LMP1 expression plasmid (pSG5_LMP1) or an empty vector (pSG5). The expression levels were measured by quantitative RT-PCR assays. Data are presented as means and SEMs. **G** Immunoblots of LMP1 and STAT4 in primary NPC cells transfected with either LMP1 expression plasmids (pSG5_LMP1) or control vectors. Protein lysates were harvested 36 h post transfection. β-Actin was used as a loading control. Uncut blots for all figures were summarized in Supplementary Figure S1

To evaluate transcriptional regulation by promoter methylation, we searched the distribution of CpG island around the promoter region of candidate genes using MethPrimer software (http://www.urogene.org/methprimer/) [25]. Results demonstrated that a dense CpG island spans the 5′-region and the first exon of CDH4 gene (Fig. 5A). To test whether the reduced expression of CDH4 was caused by promoter methylation, we treated NPC cells with 5-Aza-2′dC for demethylation. Results showed that demethylation treatment reversed expression of CDH4 in CNE1, CNE2, TW01, and TW06 NPC cells (Fig. 5B). This indicates that expression of CDH4 is at least partly regulated by epigenetic silencing.

CYLD is located on chromosome 16q12.1, a region with high frequency of DNA copy number loss in NPC [26]. We analyzed the DNA copy number changes of CYLD in NPC from the Progenetix cancer genome database (http://progenetix.org/) [27]. We found that up to 75% of NPC cases showed partial deletions of CYLD allele (Fig. 5C). We therefore examined the DNA dosage of CYLD in NPC cells by real-time PCR assays. Results revealed that loss of CYLD DNA copy number was detected in NPC-derived cell lines (0.608 ± 0.08 and 0.613 ± 0.073 for individual amplicon, respectively) as well as in primary NPC cell strains (0.852 ± 0.056 and 0.73 ± 0.04) compared to normal nasopharyngeal epithelial cell strains (NPE) or fibroblasts (Fig. 5C, D). This indicates that the suppressed CYLD expression is likely caused by a reduced DNA dosage.

EBV-encoded LMP1 has been shown to associate with activated JAK/STAT signalings in EBV-associated human malignancies [6, 28]. We therefore wanted to investigate whether LMP1 could modulate the expression of STAT4. We found that ectopic expression of LMP1 suppressed the STAT4 levels in NPC cells (Fig. 5F and G), suggesting a role of LMP1 in suppressing STAT4 expression in NPC cells.

Together, our data suggest that the expression of CDH3, CYLD, and STAT4 is, at least partially, regulated by promoter hypermethylation, DNA dosage, and EBV-encoded LMP1, respectively.

## Discussion

Nasopharyngeal carcinoma highly metastatic and its progression is affected by EBV. Because of few early warning signs and lack of NPC-specific cellular markers, most patients are diagnosed at advanced stages. In this study, we proposed a 3-gene signature, comprising two cellular molecules (CDH4 and STAT4) and one viral oncoprotein (LMP1), for early detection and differentiating advanced stage of NPC with biopsy samples. To identify predictor genes for a PCR-based diagnostic method of NPC biopsy samples, we tested several model signatures from primary NPC transcriptomic data. Logistic regression method was used to generate a predictive model for dichotomous target variable. After the cross-validation analysis, a signature model was hypothesized to be a feasible model for diagnosing NPC. This model was validated with an independent set of biopsy samples and found a 92.86% of diagnostic accuracy when a model comprising CDH4, STAT4 and LMP1 was used. Moreover, a combination of STAT3 and LMP1 had a 70.59% of prediction accuracy on stage classification. We confirmed that the mRNA transcripts of three biomarkers largely corresponded well with their protein levels in NPC tumors, supporting the applicability of the proposed classifier model for NPC early diagnosis and prediction of disease malignancy by a simple quantitative real-time RT-PCR assay, which is more sensitive and specific than that by IHC.

Although the bulk RNA sequencing data measure the average expression of a heterogenous population within NPC tumors, it remains useful for studying overall trends and differences in gene expression between NPC diagnosed with early- or late- stage of NPC. To test the applicability of our model, we tried to analyze the clinical relevance in a larger NPC dataset extracted from GEO database. A total of four NPC bulk RNA transcriptomic datasets were extracted for analysis. Among the four transcriptomic datasets, two are derived from microarray platform (GSE13597, NPC n = 25, control n = 3; and GSE12452, NPC n = 31, control n = 10) [29, 30], the other two datasets are RNA sequencing data (GSE102349, NPC n = 113; and GSE68799, NPC n = 42, control n = 4) [31]. We tried to combine multiple NPC bulk RNA transcriptome datasets to increased statistical power for our established model. However, due to deficiencies in clinical measurements and difficulty in data transformation resulting from the different platforms used to generate these data, we were unable to integrate them for validation. Eventually, GSE102349 dataset was used to validate our model since it comprises the largest sample size (n = 113) and provides relatively complete clinical measurements (survival and staging status). Using a single RNA-seq dataset has some limitations such as lack of reliable data, deficiency in clinical measurements, missing data, and insufficient sample size for statistical calculation. Hence, a comprehensive analysis on a larger NPC dataset to evaluate the applicability of these classifiers in clinical samples is needed for future study.

The three target genes identified in the current study also facilitate our understanding in the tumorigenesis of NPC. The downregulation of CYLD expression may enhance the activity of NF-κB and promote survival and oncogenic transformation of NPC. The allele loss of CYLD is in concordance with frequent LOH on 16q reported in previous allelotyping and CGH studies of NPC [26]. STAT4 belongs to the STAT families. The CTAR domains of LMP1 have been shown to activate STAT1, 3, and 5 in NPC pathogenesis [32]. STAT4 plays a critical role in IL12 response and functions in the development of the Th1 and Th2 lymphocytes, and interferon gamma signaling in response to the stimulation of cytokines [33, 34], which is important in the modulation of tumor immunity. Unlike other STAT families, we found that the LMP1 could suppress STAT4 expression in NPC cells. Consistent with our finding, IHC study by Chen et al. also shows an inactivated, cytoplasmic STAT4 in NPC [8]. Thus, it is possible that the regulation of JAK/STAT signaling in NPC pathogenesis is more complex and diverse than what we previously understood and STAT4 signaling may play a unique role associated with tumor immunity in the tumorigenesis of NPC.

EBV infection is endemic in the Southeast Asia and is detected in > 90% of NPC cases [35]. Our model comprises the EBV-encoded-LMP1, which exclusively expressed in EBV^+^-NPC tumors. It will require further analysis on EBV-negative NPC datasets to evaluate whether the proposed model can be applied to EBV-negative NPC. Collectively, the observations and knowledge identified in this work not only propose the applicability of the classifier model for NPC early diagnosis and prediction of disease malignancy, also provide insights into understanding the pathogenesis of NPC.

## Supplementary Information

Supplementary file1

Supplementary file2 (docx 28 KB)

Supplementary file3 (xlsx 89 KB)

## Data Availability

The datasets generated and analyzed during this study are included in this published article and its supplementary information files. The transcriptomic data are available in the Gene Expression Omnibus database.
