# Supplementary figures and images for: A 3-gene signature comprising CDH4, STAT4 and EBV-encoded LMP1 for early diagnosis and predicting disease progression of nasopharyngeal carcinoma
Source: Discov Oncol. 2023 Jul 1;14:119. doi: 10.1007/s12672-023-00735-x (PMC10314886; doi:10.1007/s12672-023-00735-x)

Supplementary Figure S1

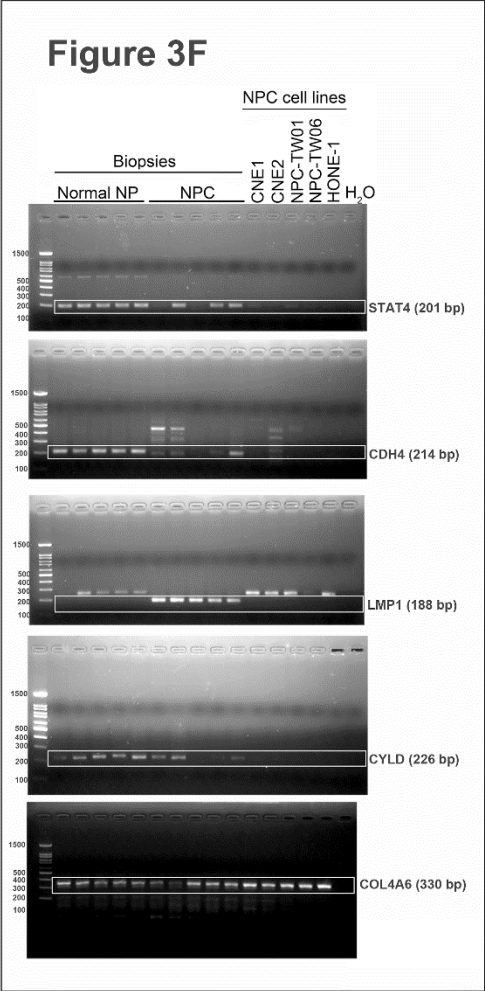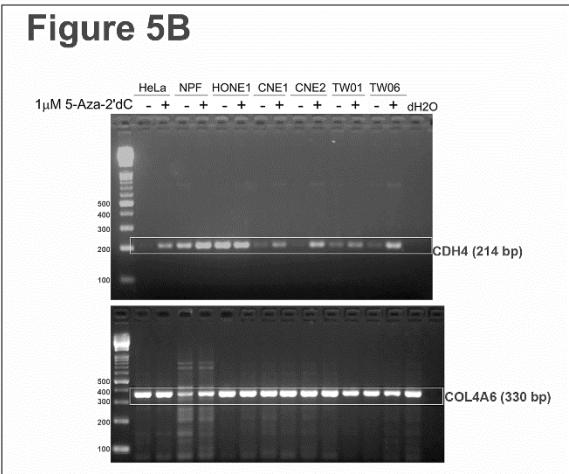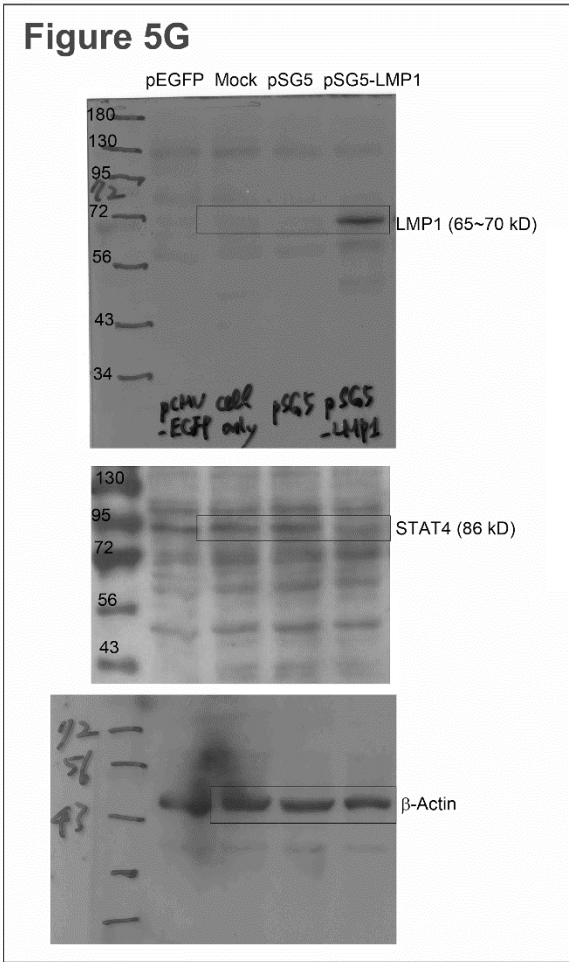

Supplementary Figure S1. Uncropped blots for figures.

Supplement: Supplementary file 1 — Supplementary file1 [file 12672_2023_735_MOESM1_ESM.pdf]
