# Supplementary material for: A 3-gene signature comprising CDH4, STAT4 and EBV-encoded LMP1 for early diagnosis and predicting disease progression of nasopharyngeal carcinoma
Source: Discov Oncol. 2023 Jul 1;14:119. doi: 10.1007/s12672-023-00735-x (PMC10314886; doi:10.1007/s12672-023-00735-x)
Supplement: Supplementary file 2 — Supplementary file2 (docx 28 KB) [file 12672_2023_735_MOESM2_ESM.docx]

| **Supplementary Table 1.** Primer information | | |
| --- | --- | --- |
| Symbol | Forward primer sequence (5'-3') | Reverse primer sequence (5'-3') |
| STAT4 | AGCCTTCGGTAAACACT | GTTGTGGGACTCAGGT |
| CDH4 | ACGGTGACAGATGTGAA | CCTCGTTGGTTACGGG |
| LMP1 | ACAAAACTGGTGGACTC | GTCTGCCCTCGTTGGA |
| PRG1 | CTTGAATCGTATCTTCCCAC | AGTCCTGGCTGTCTGA |
| CYLD | AGCTTTAAGTTAAGTGCATTGA | AGTACATCTCTAATAACTGGATTG |
| COL4A6 | AGAGGTCAGCACACAT | GCTTTACTTTGAACCAGGC |
| CYR61 | CAGGGCACACCTAGAC | CCAATCGTGGCTGCATTA |
| NBS1 | AGTAAAGATAGGGCTTCTCAG | GGCTCATTCTCAGATAGATGC |
| DSG3 | GGAGGAACCAATAAGGACT | GCAACAACCCACGGAG |
| PI3 | CCTAACCGCTGCTTGA | GAATGGACAGTGTGGGAA |
| PTGS2 | GAATGTTCCACCCGCA | ACCAGAAGGGCAGGATA |
